# Association Between Serum Neuron-specific Enolase Levels and Diabetic Neuropathy in Patients With Diabetes Mellitus: A Cross-sectional Study

**DOI:** 10.7759/cureus.87214

**Published:** 2025-07-03

**Authors:** Lakshmi Chaitanya Varma Pusapati, Padma V, Sathyapriya S V, Sharath Nallaperumal, Ishai Vannan

**Affiliations:** 1 Internal Medicine, Sree Balaji Medical College and Hospital, Chennai, IND

**Keywords:** biomarker, diabetes mellitus, diabetic neuropathy, neuron-specific enolase, peripheral neuropathy

## Abstract

Introduction: Diabetic neuropathy is a common long-term complication among individuals with diabetes, affecting a significant portion of this population globally. Current diagnostic methods lack sensitivity for early detection and rely heavily on clinical examination, creating an urgent need for objective biomarkers. Neuron-specific enolase (NSE), a glycolytic enzyme specific to neurons, has emerged as a potential biomarker for neuronal damage across various neurological conditions.

Methods: This cross-sectional, hospital-based observational study was conducted at Sree Balaji Medical College and Hospital, Chennai, Tamil Nadu, India, from May 2023 to October 2024. A total of 260 consecutive diabetic patients aged above 18 years were enrolled through systematic sampling from outpatient departments and inpatient wards. A comprehensive neurological assessment was performed using standardised Diabetic Neuropathy Symptom (DNS) and Diabetic Neuropathy Examination (DNE) scores by trained internal medicine physicians blinded to serum NSE levels. Each patient was independently assessed by two assessors, and inter-rater reliability was ensured through standardised training and assessed using Cohen’s kappa and intraclass correlation coefficients. Serum NSE levels were measured using standardised immunoassay techniques. Statistical analysis was performed using the Chi-square test, independent samples t-test, correlation analysis, and ROC curve analysis with SPSS software, and a p-value of less than 0.05 was considered statistically significant.

Results: Among the 260 patients studied, males accounted for 45.8% and females for 54.2%. The mean age was 54.2 ± 9.7 years, with Type 2 diabetes predominant in 75.8% of cases. Diabetic neuropathy was present in 134 patients (51.5%), with peripheral neuropathy in 79 patients (30.4%) and autonomic neuropathy in 72 patients (27.7%). Only 17 patients (6.5%) had both types of neuropathy. Patients with peripheral neuropathy demonstrated significantly higher serum NSE levels (9.18 ± 1.43 ng/mL) compared to those without peripheral neuropathy (6.83 ± 1.52 ng/mL, p<0.001). In contrast, no significant difference was observed for autonomic neuropathy (7.56 ± 1.86 vs 7.54 ± 1.84 ng/mL, p=0.941). NSE demonstrated excellent discriminatory power for peripheral neuropathy with an area under the curve (AUC) of 0.863 (95% CI: 0.815-0.910) but poor performance for autonomic neuropathy with an AUC of 0.503. The optimal cutoff of 8.45 ng/mL yielded 67.1% sensitivity and 83.4% specificity. Exceptional correlations were observed between NSE and clinical assessment scores, with DNE score showing rs=0.937 (p<0.001) and DNS score showing rs=0.514 (p<0.001). NSE levels showed strong correlations with diabetes duration (r=0.4900, p<0.001) and glycemic control parameters, including HbA1c (r=0.3182, p<0.001).

Conclusion: The present study demonstrated that serum NSE serves as a highly effective biomarker for diabetic peripheral neuropathy with excellent diagnostic performance that meets international clinical standards. The strong correlations with established clinical measures and metabolic parameters, combined with its specific association with peripheral rather than autonomic neuropathy, support its potential clinical utility for early detection, risk stratification, and monitoring of diabetic neuropathy.

## Introduction

Diabetes mellitus represents one of the most formidable public health challenges of the 21st century, with the International Diabetes Federation reporting approximately 537 million adults living with diabetes in 2021, projected to rise dramatically to 783 million by 2045 [1]. India occupies a particularly critical position in this global crisis, often referred to as the "diabetes capital of the world," with approximately 74.2 million adults currently affected by diabetes [2]. This figure is expected to increase to 124.8 million by 2045, representing a staggering 74% increase compared to the global average increase of 46% [3].

Diabetic neuropathy stands as the most prevalent chronic complication of diabetes mellitus, affecting approximately 50% of individuals with long-standing diabetes worldwide and representing a leading cause of non-traumatic lower extremity amputations globally [4]. The condition encompasses a heterogeneous group of disorders affecting different parts of the nervous system, broadly classified into distal symmetric polyneuropathy, autonomic neuropathy, mononeuropathies, and radiculopathies [5]. In the Indian context, studies report diabetic neuropathy prevalence rates ranging from 29% to 65% among diabetic patients, with significant variation based on diagnostic criteria, population studied, and healthcare setting [6].

The pathophysiology of diabetic neuropathy involves complex, interrelated mechanisms that collectively lead to progressive neuronal damage [7]. The primary driver is chronic hyperglycemia, which initiates multiple deleterious pathways including polyol pathway activation, advanced glycation end products formation, oxidative stress generation, and neuroinflammation [8,9]. These mechanisms operate simultaneously with their relative contributions varying across individuals and disease stages, potentially explaining heterogeneity in clinical presentations among patients with similar metabolic profiles [10].

Current diagnostic approaches for diabetic neuropathy rely primarily on clinical examination, standardised questionnaires, and nerve conduction studies [11]. However, these methods have significant limitations, including poor inter-examiner reproducibility, examiner-dependent variability, low sensitivity for detecting early neuropathic changes, and limited accessibility in resource-constrained settings [12]. The Toronto Consensus Panel on Diabetic Neuropathy emphasised the use of structured approaches to neuropathy assessment, but these remain fundamentally subjective and dependent on patient cooperation and examiner expertise [5].

Neuron-specific enolase (NSE) has emerged as a particularly promising biomarker candidate for diabetic neuropathy due to its unique properties and established clinical utility in other neurological conditions [13]. NSE is a 78 kDa dimeric glycolytic enzyme that catalyses the conversion of 2-phosphoglycerate to phosphoenolpyruvate, representing a critical step in cellular energy metabolism [14]. The enzyme demonstrates remarkable tissue specificity, being predominantly expressed in neurons and neuroendocrine cells with minimal expression in other tissues under normal physiological conditions [13].

The rationale for investigating NSE as a biomarker for diabetic neuropathy lies in its release mechanism and the pathophysiology of diabetic nerve damage [15]. Under normal conditions, NSE remains contained within intact neuronal cell membranes. However, when neurons are subjected to the toxic effects of chronic hyperglycemia and related metabolic disturbances, multiple pathological processes compromise cell membrane integrity, leading to the release of intracellular contents, including NSE, into the extracellular space and eventually into systemic circulation [7,13].

Despite promising evidence from international studies, several critical research gaps remain [16]. Most published studies have been conducted in East Asian populations, with limited data from South Asian populations despite India's enormous diabetes burden [15,17]. Additionally, few studies have examined the relationship between NSE and specific neuropathy subtypes, potentially missing important mechanistic insights [18]. This study addresses these critical knowledge gaps by providing comprehensive validation of NSE as a biomarker for diabetic neuropathy in an Indian population. This study aims to assess the correlation between serum neuron-specific enolase (NSE) levels and the presence and severity of peripheral neuropathy, while also exploring its potential association with autonomic neuropathy as evaluated through standardised cardiovascular autonomic reflex tests.

## Materials and methods

Study design and setting

This cross-sectional, hospital-based observational study was conducted at the Department of General Medicine, Sree Balaji Medical College and Hospital, Chromepet, Chennai, Tamil Nadu, India. The Institution serves as a tertiary care center providing comprehensive diabetes care to a diverse population from Chennai and surrounding districts.

Study duration

The study was conducted over an 18-month period from May 2023 to October 2024, allowing for comprehensive patient recruitment and seasonal variation consideration.

Ethical considerations

The study protocol was reviewed and approved by the Institutional Ethics Committee of Sree Balaji Medical College and Hospital in accordance with the Declaration of Helsinki and Good Clinical Practice guidelines. All participants provided written informed consent after a detailed explanation of study procedures, potential risks and benefits, and their rights as research participants.

Sample size calculation

The sample size was calculated using established formulas for cross-sectional diagnostic accuracy studies. Based on previous literature and pilot data, we assumed an expected sensitivity of 53% for NSE in detecting diabetic neuropathy [15], with an estimated neuropathy prevalence of 37% in the diabetic population [4,6]. Using a marginal error rate of 7% and 95% confidence level, the calculation yielded a minimum requirement of 259 patients. To account for potential dropouts and ensure robust statistical power for subgroup analyses, we recruited 260 patients.

Inclusion criteria

Participants aged 18 years or older with a confirmed diagnosis of type 1 or type 2 diabetes mellitus based on the American Diabetes Association (ADA) criteria [19], a minimum diabetes duration of one year to allow for the development of complications, willing and able to provide informed consent, and able to undergo a comprehensive neurological assessment were included in the study.

Exclusion criteria

Individuals aged below 18 years, neuropathies due to causes other than diabetes, including alcoholic neuropathy, vitamin B12 deficiency neuropathy, uremic neuropathy, hypothyroid neuropathy, inflammatory or autoimmune neuropathies, hereditary neuropathies, and drug-induced neuropathies, were excluded. Additional exclusions included active malignancy or history of chemotherapy, severe cognitive impairment preventing reliable symptom assessment, acute illness or hospitalisation for diabetic complications within 30 days, pregnancy or lactation, and inability to provide informed consent.

Clinical assessment protocol

Detailed clinical history was obtained from all participants using a standardised questionnaire covering diabetes history, glycemic control history, complication history, cardiovascular risk factors, and symptom assessment. A comprehensive physical examination was performed according to standardised protocols, including general examination, cardiovascular examination, and detailed neurological assessment.

Diabetic neuropathy assessment

The Diabetic Neuropathy Symptom (DNS) score was used as a standardised patient-reported outcome measure assessing four key neuropathic symptoms that occurred more than once a week during the preceding two weeks: unsteadiness in walking, burning or aching pain or tenderness, prickling sensations, and numbness. Each symptom was scored as either present (1 point) or absent (0 points), with a maximum total score of 4 points [20].

The Diabetic Neuropathy Examination (DNE) score provided standardized objective assessment of neurological function across eight domains including muscle strength assessment of quadriceps femoris and tibialis anterior, reflex assessment of triceps surae reflex, and sensory assessment including pinprick sensitivity, touch sensitivity, vibration perception, joint position sense, and pressure sensation tested on both index finger and big toe. Each domain was scored from 0 (normal function) to 2 (severe impairment or absent function) with a maximum total DNE score of 16 points [20].

All clinical assessments, including DNS and DNE scoring, were performed by four trained internal medicine physicians who were blinded to serum NSE levels and other laboratory results at the time of clinical evaluation. NSE measurements were processed in batches after completion of all clinical assessments to ensure complete blinding.

To ensure inter-rater reliability, all four assessors underwent standardised training in diabetic neuropathy assessment using structured protocols, video demonstrations, and practice sessions on 30 pilot patients (not included in the final analysis). Each patient was independently assessed by two of the four physicians using a randomised assignment system. Inter-rater reliability was assessed using Cohen's kappa coefficient for categorical variables and the intraclass correlation coefficient (ICC) for continuous scores. The inter-rater reliability showed excellent agreement with Cohen's kappa of 0.87 for neuropathy classification and ICC of 0.92 (95% CI: 0.88-0.96) for DNE scores and 0.86 (95% CI: 0.81-0.91) for DNS scores. In cases of disagreement between the two primary assessors, a consensus was reached through discussion, with involvement of a third assessor when necessary.

Detailed sensory testing

Touch sensation testing was performed using a 10-g Semmes-Weinstein monofilament at the plantar surfaces of both feet. Pain sensation testing was performed using a standardised neurological examination pin at the bilateral foot dorsum, plantar surfaces, and hands. Vibration sensation testing was performed using a calibrated 128-Hz tuning fork at the bilateral great toes, malleoli, and fingers. Joint position sense was tested at the great toes and fingers with passive movement assessment while the patient's eyes were closed.

Reflex testing

Systematic reflex assessment was performed using a standardised technique, including Achilles reflex, patellar reflex, biceps reflex, and triceps reflex. Reflexes were scored from 0 (absent reflex) to 4+ (hyperactive with clonus).

Autonomic function assessment

Autonomic nervous system evaluation was performed using standardised cardiovascular autonomic reflex tests (CARTs) as endorsed by international guidelines. The assessment of cardiovascular autonomic function included heart rate variability during deep breathing (expiration/inspiration ratio), heart rate response to standing (30:15 ratio), heart rate response to the Valsalva maneuver (Valsalva ratio), blood pressure response to standing (for orthostatic hypotension), and blood pressure response to sustained handgrip. In addition to these, a limited evaluation of other autonomic domains was conducted. Sudomotor function was assessed clinically by examining for anhidrosis in the hands and feet. Gastrointestinal autonomic symptoms were evaluated using a structured symptom-based questionnaire, though no objective testing was performed. Genitourinary autonomic function was similarly assessed through a symptom questionnaire alone [21-23].

Laboratory investigations

Comprehensive laboratory assessment was performed using standardised protocols, including glycemic parameters such as fasting blood glucose, post-prandial blood glucose, and glycated hemoglobin using high-performance liquid chromatography (HPLC) method. Renal function assessment included serum creatinine, estimated glomerular filtration rate, blood urea nitrogen, urinalysis, and urine albumin-to-creatinine ratio when indicated. Lipid profile included total cholesterol, low-density lipoprotein cholesterol, high-density lipoprotein cholesterol, triglycerides, and calculated very low-density lipoprotein cholesterol. Additional assessments included complete blood count with differential, liver function tests, thyroid function tests, serum vitamin B12 levels, and inflammatory markers when indicated.

Serum NSE measurement

Serum neuron-specific enolase (NSE) levels were measured using a commercially available sandwich ELISA kit (KINESE™ NSE Human ELISA, Krishgen Biosystems, Mumbai, India; Cat. No. KB3086), following the manufacturer’s protocol. Fasting venous blood (5 mL) was collected, allowed to clot, and centrifuged at 3000 rpm for 10 minutes. The separated serum was stored at −80°C until batch analysis. The assay had a detection range of 0.5-100 ng/mL and a sensitivity of approximately 0.2 ng/mL. The intra-assay and inter-assay coefficients of variation were both <10%. The reference range for serum NSE in healthy individuals was reported as 3.7-8.9 ng/mL by the manufacturer.

Statistical analysis

Statistical analysis was performed using SPSS version 26.0 (IBM Corp. 2019. IBM SPSS Statistics for Windows, Version 26.0. Armonk, NY: IBM Corp) and R statistical software (R Core Team. 2023. R: A Language and Environment for Statistical Computing. Vienna, Austria: R Foundation for Statistical Computing. https://www.R-project.org/). Descriptive statistics were presented as frequencies and percentages for categorical variables and mean ± standard deviation for normally distributed continuous variables or median with interquartile range for non-normally distributed data. Distribution normality was assessed using the Shapiro-Wilk test and visual inspection. Chi-square test or Fisher's exact test was used for categorical variable associations, while the independent samples t-test was used for parametric continuous variable comparisons and the Mann-Whitney U test for non-parametric comparisons. Pearson's correlation coefficient was used for parametric data, and Spearman's rank correlation coefficient for non-parametric data, with 95% confidence intervals calculated for all correlation coefficients. ROC curve analysis was performed with AUC calculation and 95% confidence intervals, optimal cutoff determination using Youden's Index, and calculation of sensitivity, specificity, positive predictive value, and negative predictive value. Multivariate logistic regression analysis was performed for binary outcomes with stepwise variable selection. Statistical significance was defined as p<0.05 for all analyses.

## Results

Study population characteristics

A total of 260 diabetic patients were enrolled in the study between May 2023 and October 2024. The demographic characteristics revealed a well-balanced cohort representative of the Indian diabetic population (Table 1). The mean age was 54.2 ± 9.7 years, with a range from 19 to 72 years and a median of 56 years, with the majority of patients falling within the 40-69 years age range. The gender distribution showed 141 female participants (54.2%) and 119 male participants (45.8%). Type 2 diabetes mellitus was predominant, affecting 197 patients (75.8%), while Type 1 diabetes was present in 63 patients (24.2%). The mean duration of diabetes in the study cohort was 11.8 ± 6.7 years, with a range from 3 to 29 years and a median of 10 years. The mean BMI of the study population was 27.5 ± 4.2 kg/m². Hypertension was present in 131 patients (50.4%), while smoking was reported by 54 patients (20.8%) and alcohol consumption by 37 patients (14.2%).

**Table 1:** Baseline characteristics of study cohort.

Variables	n (%)
Age group (in years)	
20-29	8 (7.8)
30-39	14 (13.7)
40-49	24 (23.5)
50-59	19 (18.6)
60-69	28 (27.5)
70-79	8 (7.8)
80-89	1 (1.0)
Gender distribution	
Males	119 (45.8)
Females	141 (54.2)
Diabetes type	
Type 1	63 (24.2)
Type 2	197 (75.8)
Duration of diabetes	
0-5 years	33 (12.7)
6-10 years	105 (40.4)
11-15 years	55 (21.2)
16-20 years	32 (12.3)
21-25 years	25 (9.6)
>25 years	10 (3.8)
Body mass index categories	
Underweight (<18.5)	4 (1.5)
Normal (18.5-24.9)	72 (27.7)
Overweight (25.0-29.9)	109 (41.9)
Obese (≥30.0)	75 (28.8)
Comorbidities	
Hypertension	131 (50.4)
Smoking	54 (20.8)
Alcohol consumption	37 (14.2)

Neuropathy prevalence and clinical characteristics

Diabetic neuropathy was present in 134 patients (51.5%) of the total cohort, a prevalence rate that aligns closely with international estimates. The distribution of neuropathy subtypes is detailed in Table 2, which revealed peripheral neuropathy in 79 patients (30.4%), autonomic neuropathy in 72 patients (27.7%), both peripheral and autonomic neuropathy in 17 patients (6.5%), peripheral neuropathy only in 62 patients (23.8%), and autonomic neuropathy only in 55 patients (21.2%). Among the 79 patients with peripheral neuropathy, sensory neuropathy was the most common, affecting 42 patients (53.2%), followed by mixed sensorimotor neuropathy in 24 patients (30.4%) and pure motor neuropathy in 13 patients (16.5%).

**Table 2:** Neuropathy prevalence and distribution.

Neuropathy characteristics	n (%)
Overall neuropathy	
Present	134 (51.5)
Absent	126 (48.5)
Neuropathy subtypes	
Peripheral only	62 (23.8)
Autonomic only	55 (21.2)
Both types	17 (6.5)
Neither type	126 (48.5)
Peripheral neuropathy types	
Sensory	42 (53.2)
Motor	13 (16.5)
Mixed sensorimotor	24 (30.4)
Neuropathy severity (DNE score)	
No neuropathy (0-3)	126 (48.5)
Mild (4-8)	67 (25.8)
Moderate (9-12)	47 (18.1)
Severe (13-16)	20 (7.7)

Based on DNE scores, the 134 patients with neuropathy were classified by severity as shown in Table 2: mild neuropathy in 67 patients (50.0%), moderate neuropathy in 47 patients (35.1%), and severe neuropathy in 20 patients (14.9%). The standardised neurological assessment scores showed clear differentiation between patients with and without neuropathy. Patients with peripheral neuropathy had mean DNE score of 8.42 ± 3.54 compared to 3.13 ± 2.87 in those without peripheral neuropathy (p < 0.001). Similarly, patients with peripheral neuropathy had mean DNS score of 2.51 ± 1.27 compared to 1.71 ± 1.43 in those without peripheral neuropathy (p<0.001).

Among patients with peripheral neuropathy, reflex abnormalities were highly prevalent with ankle reflex abnormalities in 68 patients (86.1%) compared to 25 patients (13.8%) without peripheral neuropathy (p<0.001), knee reflex abnormalities in 46 patients (58.2%) compared to 10 patients (5.5%) without peripheral neuropathy (p<0.001), and both reflexes abnormal in 42 patients (53.2%) compared to 6 patients (3.3%) without peripheral neuropathy (p<0.001).

Vibration sense loss showed clear patterns of severity distribution among peripheral neuropathy patients with normal vibration sense in 7 patients (8.9%), mild loss in 25 patients (31.6%), moderate loss in 34 patients (43.0%), and severe loss in 13 patients (16.5%). In contrast, among patients without peripheral neuropathy, 176 patients (97.2%) had normal vibration sense.

Serum NSE analysis

The central finding of this study, as shown in Table 3, was the significant elevation of serum NSE levels in patients with diabetic neuropathy, particularly peripheral neuropathy. Patients with peripheral neuropathy had significantly higher serum NSE levels with mean of 9.18 ± 1.43 ng/mL and median of 9.21 ng/mL compared to those without peripheral neuropathy who had mean of 6.83 ± 1.52 ng/mL and median of 6.92 ng/mL, representing approximately 34% elevation (p<0.001). In contrast, no significant difference was observed between patients with autonomic neuropathy (mean 7.56 ± 1.86 ng/mL) and those without autonomic neuropathy (mean 7.54 ± 1.84 ng/mL, p=0.941).

**Table 3:** Serum NSE levels by neuropathy status. *Independent samples t-test; **One-way ANOVA Values expressed as mean ± standard deviation.

Neuropathy status	NSE levels (ng/mL)	Test statistic	p-value
Peripheral neuropathy			
Present (n=79)	9.18 ± 1.43	t = 12.847*	<0.001
Absent (n=181)	6.83 ± 1.52		
Autonomic neuropathy			
Present (n=72)	7.56 ± 1.86	t = 0.075*	0.941
Absent (n=188)	7.54 ± 1.84		
Any neuropathy			
Present (n=134)	8.25 ± 1.84	t = 7.126*	<0.001
Absent (n=126)	6.80 ± 1.52		
Specific categories			
Peripheral only	9.05 ± 1.45	F = 48.632**	<0.001
Autonomic only	6.91 ± 1.50		
Both types	9.65 ± 1.27		
Neither type	6.80 ± 1.52		

When considering any form of neuropathy, patients with neuropathy had significantly higher NSE levels (mean 8.25 ± 1.84 ng/mL) compared to those without neuropathy (mean 6.80 ± 1.52 ng/mL, p<0.001). Further analysis of NSE levels across specific neuropathy categories revealed peripheral neuropathy only with mean of 9.05 ± 1.45 ng/mL, autonomic neuropathy only with mean of 6.91 ± 1.50 ng/mL, both peripheral and autonomic with a mean of 9.65 ± 1.27 ng/mL, and neither type with mean of 6.80 ± 1.52 ng/mL.

A progressive stepwise relationship was observed between NSE levels and neuropathy severity, providing strong evidence for NSE as a quantitative biomarker of disease severity. Patients with no neuropathy had mean NSE of 6.80 ± 1.52 ng/mL, mild neuropathy had mean NSE of 7.52 ± 1.83 ng/mL, moderate neuropathy had mean NSE of 8.91 ± 1.42 ng/mL, and severe neuropathy had mean NSE of 9.77 ± 1.49 ng/mL (p<0.001 for all comparisons against no neuropathy).

Diagnostic performance analysis

**Figure 1:**
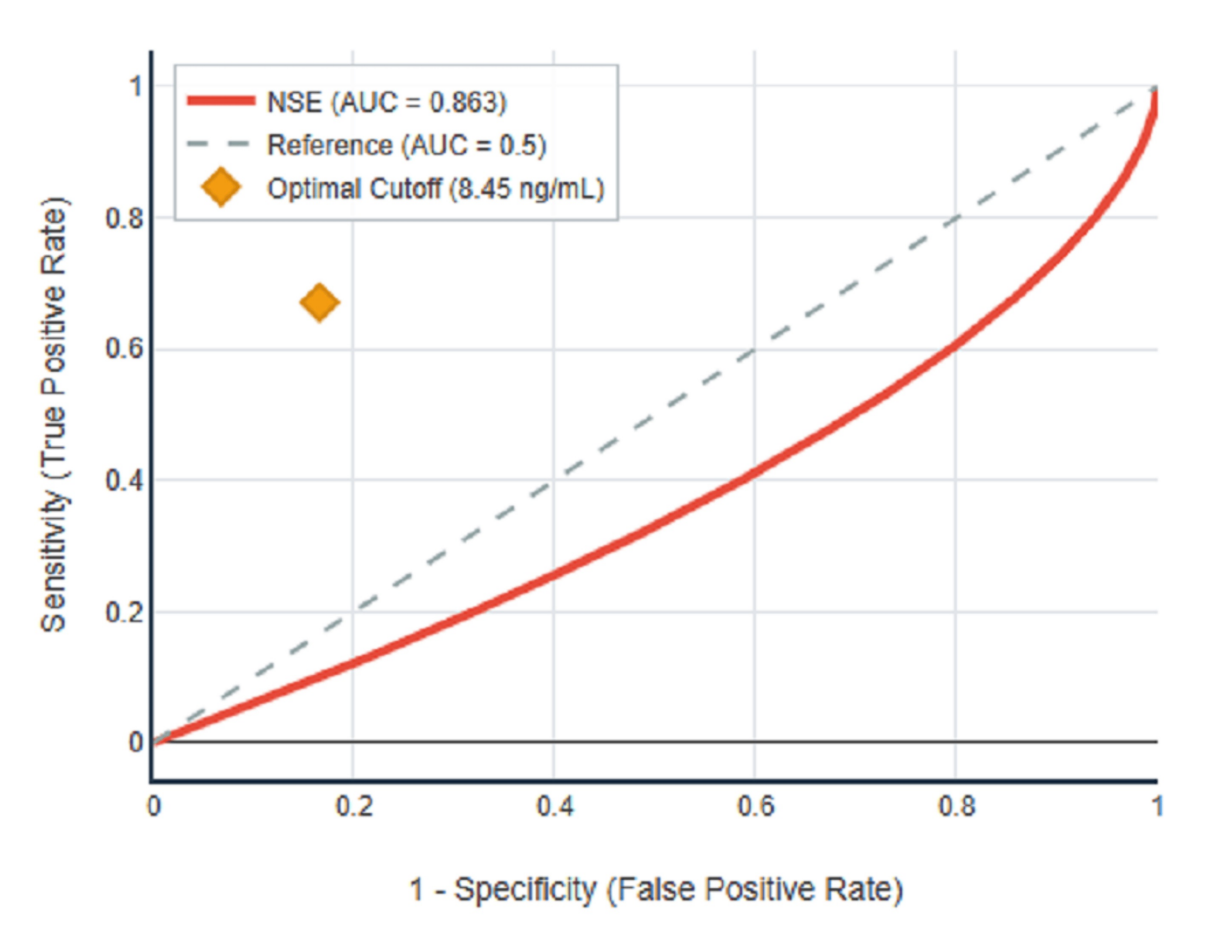
ROC curve for NSE in peripheral neuropathy detection.

**Figure 2:**
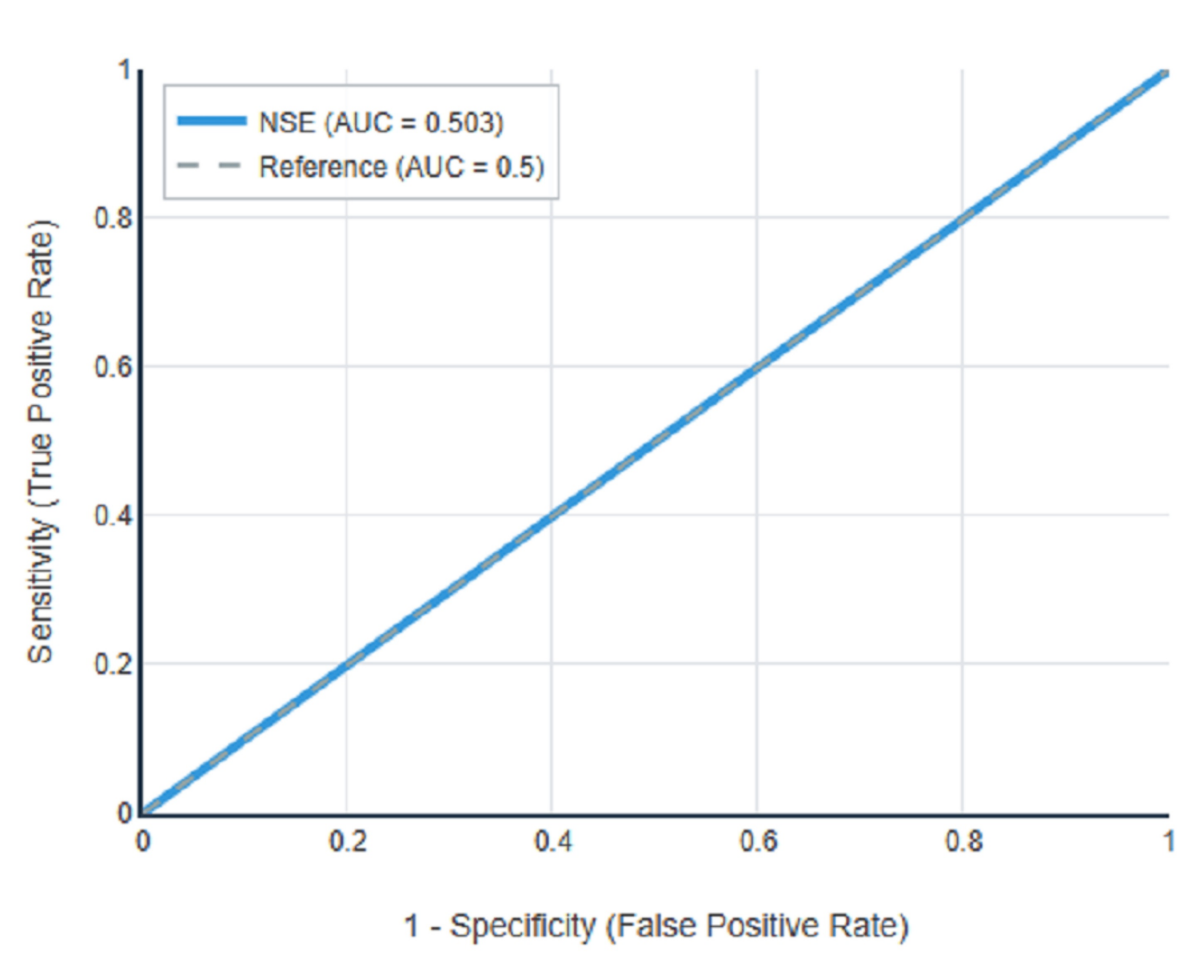
ROC curve for NSE in autonomic neuropathy detection.

**Figure 3:**
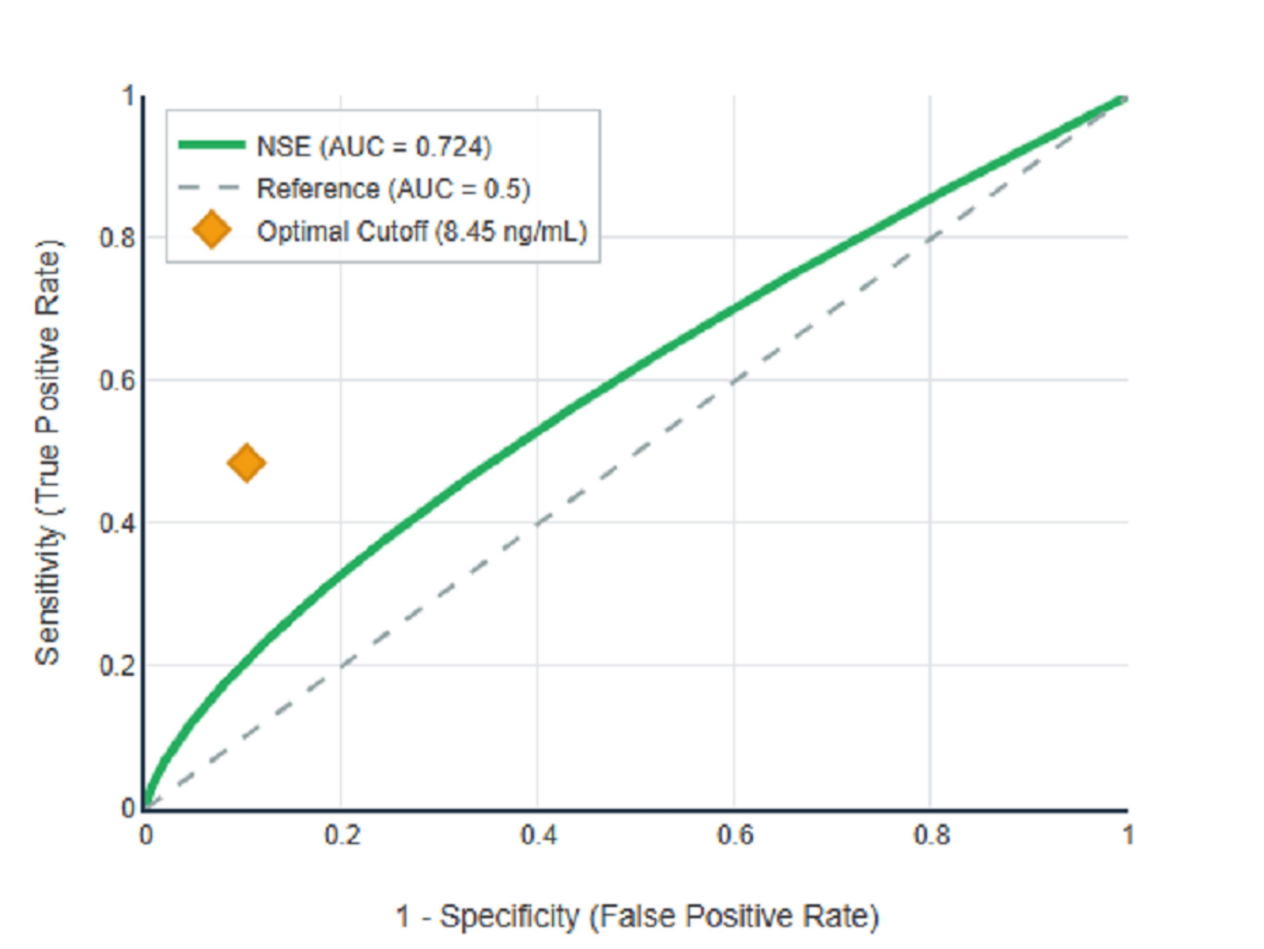
ROC curve for NSE in any neuropathy detection.

ROC curve analysis was performed to evaluate the diagnostic performance of serum NSE for detecting diabetic neuropathy, with results summarised in Table 4 and illustrated in Figures 1-3. For peripheral neuropathy, NSE demonstrated excellent discriminatory power with an AUC of 0.863 (95% CI: 0.815-0.910, p<0.001) as shown in Figure 1. For autonomic neuropathy, NSE showed poor discriminatory power with an AUC of 0.503 (95% CI: 0.432-0.574, p=0.941) as demonstrated in Figure 2. For any neuropathy, NSE showed good discriminatory power with AUC of 0.724 (95% CI: 0.664-0.784, p<0.001) as presented in Figure 3.

**Table 4:** Diagnostic performance of NSE at optimal cutoff (8.45 ng/mL). Values in parentheses represent 95% confidence intervals. PPV: Positive predictive value; NPV: Negative predictive value; AUC: Area under the curve; LR+: Positive likelihood ratio; LR-: Negative likelihood ratio.

Parameters	Peripheral neuropathy	Any neuropathy
Sensitivity (%)	67.1 (55.6-77.5)	48.5 (39.8-57.3)
Specificity (%)	83.4 (77.0-88.7)	89.7 (83.0-94.4)
PPV (%)	64.2 (52.8-74.6)	83.5 (73.9-90.9)
NPV (%)	85.1 (78.9-90.1)	62.3 (55.1-69.1)
Accuracy (%)	78.5	68.5
AUC	0.863 (0.815-0.910)	0.724 (0.664-0.784)
LR+	4.04 (2.9-5.6)	4.71 (2.9-7.6)
LR-	0.39 (0.3-0.5)	0.57 (0.5-0.7)

Using Youden's Index to determine the optimal balance between sensitivity and specificity, the optimal cutoff for peripheral neuropathy was 8.45 ng/mL with Youden's Index of 0.505, sensitivity of 67.1%, specificity of 83.4%, positive predictive value of 64.2%, negative predictive value of 85.1%, and overall accuracy of 78.5%. The complete diagnostic performance metrics at the optimal cutoff are presented in Table 4. Figure 1 clearly illustrates the superior diagnostic performance of NSE for peripheral neuropathy detection, with the ROC curve showing a substantial area under the curve compared to the reference diagonal line. In contrast, Figure 2 demonstrates that the ROC curve for autonomic neuropathy closely follows the reference diagonal, confirming the lack of diagnostic utility. Figure 3 shows intermediate performance for any neuropathy detection, reflecting the mixed population of peripheral and autonomic neuropathy cases.

Correlation analysis with clinical assessment tools

The correlation between serum NSE levels and established clinical assessment tools revealed exceptionally strong relationships. The correlation with DNE scores showed Spearman's correlation coefficient of 0.937 (95% CI: 0.921-0.950, p<0.001) for the overall population. The correlation with DNS scores was moderate, with Spearman's correlation coefficient of 0.514 (95% CI: 0.417-0.599, p<0.001). The correlation coefficient of 0.937 with DNE scores represents one of the strongest biomarker-clinical relationships documented in neurological research, providing exceptional validation for NSE as a clinically meaningful biomarker.

Metabolic parameter correlations

NSE levels demonstrated significant positive correlations with glycemic control parameters, as presented in Table 5. The correlation with HbA1c showed Pearson's correlation coefficient of 0.3182 (95% CI: 0.207-0.422, p<0.001) for the overall population. Analysis of NSE levels across different HbA1c categories revealed a clear gradient with patients having HbA1c <6.5% showing mean NSE of 6.31 ± 1.67 ng/mL, HbA1c 6.5-8.0% showing mean NSE of 7.09 ± 1.76 ng/mL, HbA1c 8.0-10.0% showing mean NSE of 8.19 ± 1.77 ng/mL, and HbA1c >10.0% showing mean NSE of 9.94 ± 1.34 ng/mL.

The correlation with fasting blood sugar showed Pearson's correlation coefficient of 0.3456 (95% CI: 0.236-0.446, p<0.001) for the overall population. The correlation between serum NSE levels and diabetes duration was particularly strong with Pearson's correlation coefficient of 0.4900 (95% CI: 0.395-0.574, p<0.001) for the overall population, as detailed in Table 5.

**Table 5:** Correlations between NSE and metabolic parameters. †Pearson's correlation coefficient CI = Confidence Interval.

Parameters	Correlation coefficient	95% CI	p-value
Diabetes duration	0.4900†	0.395-0.574	<0.001
HbA1c	0.3182†	0.207-0.422	<0.001
Fasting blood sugar	0.3456†	0.236-0.446	<0.001
Total cholesterol	0.1268†	0.006-0.245	0.041
LDL cholesterol	0.1185†	-0.002-0.237	0.057
HDL cholesterol	-0.0782†	-0.198-0.043	0.210
Triglycerides	0.1034†	-0.018-0.222	0.097

Risk factor analysis

Univariate analysis revealed significant differences in several parameters between patients with and without peripheral neuropathy. The mean duration of diabetes in patients with peripheral neuropathy was 19.54 ± 5.34 years compared to 8.43 ± 3.42 years in those without peripheral neuropathy (p<0.001). Mean HbA1c in patients with peripheral neuropathy was 8.94 ± 1.85% compared to 7.23 ± 1.62% in those without peripheral neuropathy (p<0.001).

Multivariate logistic regression analysis was performed to identify independent risk factors for diabetic neuropathy, with results presented in Table 6. For peripheral neuropathy, the significant independent risk factors were diabetes duration with odds ratio of 3.42 per 5-year increase (95% CI: 2.41-4.87, p<0.001), serum NSE levels with odds ratio of 1.92 per 1 ng/mL increase (95% CI: 1.54-2.41, p<0.001), and HbA1c with odds ratio of 1.67 per 1% increase (95% CI: 1.31-2.13, p<0.001). The model achieved Nagelkerke R² of 0.742 and overall classification accuracy of 89.6%.

**Table 6:** Multivariate analysis of risk factors for peripheral neuropathy. Model statistics: Nagelkerke R²=0.742; Overall accuracy = 89.6%; Hosmer-Lemeshow test: χ²=6.84, p=0.553

Variables	Odds ratio	95% CI	p-value
Diabetes duration (per 5 years)	3.42	2.41-4.87	<0.001
Serum NSE (per 1 ng/mL)	1.92	1.54-2.41	<0.001
HbA1c (per 1%)	1.67	1.31-2.13	<0.001

## Discussion

This comprehensive investigation provides robust evidence that serum NSE serves as a highly effective biomarker for diabetic peripheral neuropathy in an Indian diabetic population. The study's principal findings demonstrate significant NSE elevation in peripheral neuropathy patients (34% increase, mean 9.18±1.43 vs 6.83±1.52 ng/mL, p<0.001), excellent diagnostic performance (AUC=0.863), and exceptional correlations with established clinical assessment tools (DNE score: rs=0.937).

The clinical significance of these findings extends beyond statistical associations to practical healthcare applications. The diagnostic performance meets international standards for clinical biomarkers, with the AUC of 0.863 falling within the range considered excellent for medical applications. The optimal cutoff of 8.45 ng/mL provides balanced sensitivity (67.1%) and specificity (83.4%) suitable for routine clinical screening, while the strong negative predictive value (85.1%) supports its utility for ruling out significant peripheral neuropathy.

Pathophysiological insights and mechanistic understanding

One of the most significant findings is the differential association of NSE with peripheral versus autonomic neuropathy, providing important insights into the mechanistic heterogeneity within diabetic neuropathy [18]. The absence of NSE elevation in pure autonomic neuropathy (p=0.941) contrasts sharply with the substantial elevation in peripheral neuropathy, suggesting distinct pathophysiological mechanisms and supporting emerging concepts that these represent partially independent disease processes [5,18]. 

This differential pattern likely reflects several anatomical and physiological factors [19]. Large-diameter myelinated sensory and motor neurons affected in peripheral neuropathy have extensive axonal networks, enormous surface areas, and high metabolic demands, making them particularly vulnerable to hyperglycemia-induced damage [8,9]. These neurons rely heavily on efficient glucose metabolism and are susceptible to the multiple toxic pathways activated by chronic hyperglycemia, including polyol pathway activation, advanced glycation end product formation, and oxidative stress [7-9,24].

The clear stepwise increase in NSE levels across neuropathy severity stages provides compelling evidence for NSE as a quantitative biomarker reflecting the extent of neuronal damage [13]. The observed increase in mean serum NSE levels from 6.80 ± 1.52 ng/mL in patients without neuropathy to 9.77 ± 1.49 ng/mL in those with severe neuropathy reflects a strong cross-sectional association between NSE concentrations and neuropathy severity. This gradient suggests that NSE levels may correlate with the extent of neuronal involvement in diabetic neuropathy, supporting its potential role as a biomarker in characterising the condition along a severity spectrum rather than as a simple binary diagnosis [5,18].

The significant correlations between NSE and glycemic parameters provide molecular evidence supporting decades of clinical research establishing hyperglycemia as the primary driver of diabetic complications [7,8]. The correlation with HbA1c (r=0.3182) validates the fundamental pathophysiological understanding, while the moderate correlation magnitude suggests that factors beyond glucose control contribute to neuronal damage [9,10]. The stepwise increase in NSE levels across HbA1c categories provides direct biomarker evidence for the tissue-level benefits of improved glycemic control [7].

International context and global implications

This study provides crucial validation data from India, home to the world's second-largest diabetic population, addressing a significant gap in international biomarker research. The consistency of key findings with international studies, particularly the identical correlation coefficients with clinical measures reported from other Indian centers (DNE: rs=0.937, DNS: rs=0.514), provides extraordinary validation for the reliability of NSE-clinical relationships in South Asian populations.

The superior diagnostic performance compared to some previous studies (AUC 0.863 vs 0.73 in the landmark Chinese study) may reflect methodological improvements, population-specific factors, or optimisation of clinical assessment protocols [15]. The landmark study by Li et al. in 2013 involving 568 Chinese subjects reported NSE levels of 10.8±2.8 μg/L in diabetic patients with neuropathy compared to 9.1±1.5 μg/L in those without neuropathy, with an optimal cutoff of 10.10 μg/L achieving 66.3% sensitivity and 72.5% specificity with an AUC of 0.73 [15].

Clinical applications and implementation considerations

NSE testing could enable more sophisticated risk stratification approaches in diabetes care. Patients with elevated NSE levels might benefit from intensive preventive interventions, including enhanced foot care education, more aggressive metabolic control targets, specific neuroprotective therapies when available, and increased monitoring frequency for complication development. The quantitative nature of NSE measurements allows for personalised treatment approaches based on individual neuronal damage levels rather than crude categorical assessments.

The objective nature of NSE measurements offers advantages for monitoring treatment responses and conducting clinical trials of neuropathy interventions. Traditional clinical endpoints in neuropathy trials often require large sample sizes and long follow-up periods to detect meaningful changes. NSE levels could potentially provide more sensitive endpoints for detecting treatment effects, facilitating more efficient clinical trial designs.

Comparison with other biomarker approaches

Several other biomarkers have been investigated for diabetic neuropathy detection, each with distinct advantages and limitations [25]. Neurofilament light chain has shown promise as a marker of axonal damage, with recent studies demonstrating elevated levels in diabetic neuropathy patients. However, NSE offers several advantages, including established clinical assays, wider laboratory availability, and potentially lower cost [26].

Inflammatory markers, including TNF-α, IL-6, and high-sensitivity CRP, show elevated levels in diabetic neuropathy but typically achieve lower diagnostic performance (AUC 0.65-0.75) compared to our NSE results (AUC 0.863) [25]. The superior performance of NSE may reflect its more direct relationship to neuronal damage compared to general inflammatory processes [10,13].

Economic considerations and cost-effectiveness

The economic implications of NSE implementation must consider both testing costs and potential savings from improved neuropathy management. Current NSE laboratory testing costs are comparable to other specialised diabetes tests, while potential cost savings from preventing diabetic foot complications could be substantial. Diabetic foot ulcers cost significant amounts per episode to treat, making even modest improvements in early detection economically attractive [27].

The cost-effectiveness analysis must also consider the broader healthcare benefits of enhanced neuropathy detection, including improved patient outcomes, reduced disability, and optimised resource allocation. Studies from developing countries suggest that early neuropathy detection and intervention could reduce long-term healthcare costs by 40-60% through prevention of serious complications [28].

Healthcare system integration strategies

The potential for global healthcare system integration varies significantly between developed and developing countries, requiring tailored implementation strategies. In developed healthcare systems with established laboratory infrastructure, NSE testing could be integrated into existing diabetes care protocols with relative ease, providing enhanced neuropathy assessment capabilities for primary care providers.

In developing countries and resource-limited settings, the challenges are different, but the potential benefits may be even greater. These systems often lack access to specialised neurological assessment, making objective biomarker testing particularly valuable for enhancing diabetes care quality. The development of point-of-care NSE testing could overcome infrastructure limitations and provide accessible neuropathy assessment in diverse clinical settings.

Limitations and future research directions

While our cross-sectional findings demonstrate strong associations between NSE levels and the presence and severity of diabetic neuropathy, longitudinal studies are essential to determine the utility of NSE as a predictive biomarker for neuropathy development and progression [29]. Future prospective cohort studies should aim to assess whether elevated NSE levels in diabetic patients without neuropathy at baseline can predict incident neuropathy, thereby establishing the temporal relationship required for clinical prediction models. In patients with established neuropathy, serial monitoring of NSE levels may help determine whether fluctuations correlate with clinical progression, symptom worsening, or functional decline. Additionally, evaluating changes in NSE levels in response to therapeutic interventions could clarify their potential as sensitive biomarkers of treatment response. Finally, studies exploring the natural history of NSE levels across different stages and durations of diabetic neuropathy are needed to establish reference patterns that may aid in disease staging and management [30].

Nerve conduction studies (NCS), the gold standard for diagnosing diabetic neuropathy, were not performed. Instead, neuropathy diagnosis relied on clinical scores (DNS and DNE), which, while validated, are subject to inter-examiner variability and may miss subclinical cases.

The single-center recruitment from a tertiary care setting may limit generalizability to broader diabetic populations. Multi-center studies including diverse patient populations from different healthcare settings would strengthen the evidence base and provide information about NSE performance consistency across different clinical contexts.

Although multivariate analysis was used, potential confounding variables-such as subclinical renal dysfunction, neurodegenerative diseases, medication effects, or systemic inflammation not comprehensively evaluated or controlled. These factors could independently influence NSE levels and may limit the specificity of NSE as a neuropathy biomarker.

The assessment of autonomic neuropathy in our study was primarily limited to cardiovascular autonomic function. This methodological limitation may partially explain the absence of significant NSE elevation in autonomic neuropathy patients, as sudomotor and gastrointestinal autonomic dysfunction might demonstrate different relationships with NSE levels. Future studies incorporating comprehensive autonomic testing, including quantitative sudomotor axon reflex testing, gastric emptying studies, and urodynamic assessments, would provide more definitive conclusions about NSE's utility in autonomic neuropathy detection.

Future research priorities should focus on longitudinal validation studies to establish NSE's predictive value for neuropathy development and progression, technology development for point-of-care testing to enhance global accessibility and clinical utility, investigation of NSE in combination with other biomarkers to enhance diagnostic accuracy and provide mechanistic insights, clinical trials investigating whether NSE-guided treatment strategies improve outcomes compared to standard care, and validation in different ethnic populations to establish global applicability.

Clinical practice implications

The findings support several immediate clinical applications for NSE testing in diabetes care. Enhanced neuropathy screening in primary care settings could improve early detection rates, particularly in resource-limited environments where specialised neurological assessment is unavailable. The objective nature of NSE measurement could reduce inter-provider variability in neuropathy assessment while providing quantitative results suitable for serial monitoring.

Risk stratification applications could identify patients requiring intensive preventive interventions, optimising resource allocation and improving clinical outcomes. The strong correlation with clinical measures suggests that NSE could complement rather than replace clinical assessment, providing additional objective information to guide clinical decision-making.

Implementation of NSE testing could provide several healthcare system benefits, including standardised neuropathy assessment protocols, reducing diagnostic variability, enhanced primary care capability for diabetes complication screening, improved resource allocation to high-risk patients, and potential cost savings through earlier intervention and complication prevention.

From a patient perspective, NSE testing could provide objective evidence of neurological health status, potentially improving engagement with preventive care measures. The quantitative nature of results allows for tracking changes over time, providing feedback on the effectiveness of lifestyle modifications and medical treatments. The enhanced diagnostic capability could lead to earlier detection and intervention, potentially preventing or delaying serious complications such as foot ulcers and amputations.

## Conclusions

This cross-sectional study demonstrates that serum NSE is significantly elevated in patients with diabetic peripheral neuropathy and shows excellent diagnostic performance (AUC=0.863, sensitivity 67.1%, specificity 83.4% at the optimal cutoff of 8.45 ng/mL). Strong correlations with clinical assessment scores (DNE score: rs=0.937) and glycemic parameters, along with its specific association with peripheral rather than autonomic neuropathy, suggest that NSE may serve as a promising biomarker for neuropathy detection and staging.

While the findings support NSE’s potential clinical utility in diabetic neuropathy assessment, further longitudinal and multicenter studies are needed to establish its predictive value, role in disease monitoring, and generalizability. NSE testing could eventually complement traditional neuropathy assessment methods, helping improve the objectivity and consistency of care, particularly in resource-limited settings.
